# Changes in Serine Racemase-Dependent Modulation of NMDA Receptor: Impact on Physiological and Pathological Brain Aging

**DOI:** 10.3389/fmolb.2018.00106

**Published:** 2018-11-28

**Authors:** Jean-Marie Billard

**Affiliations:** UNICAEN, INSERM, COMETE, Normandie University, Caen, France

**Keywords:** NMDA receptors, serine racemase, aging, Alzheimer's disease, **d**-serine, long term potentiation, glutamate

## Abstract

The N-methyl-D-Aspartate glutamate receptors (NMDARs) are pivotal for the functional and morphological plasticity that are required in neuronal networks for efficient brain activities and notably for cognitive-related abilities. Because NMDARs are heterogeneous in subunit composition and associated with multiple functional regulatory sites, their efficacy is under the tonic influence of numerous allosteric modulations, whose dysfunction generally represents the first step generating pathological states. Among the enzymatic candidates, serine racemase (SR) has recently gathered an increasing interest considering that it tightly regulates the production of d-serine, an amino acid now viewed as the main endogenous co-agonist necessary for NMDAR activation. Nowadays, SR deregulation is associated with a wide range of neurological and psychiatric diseases including schizophrenia, amyotrophic lateral sclerosis, and depression. This review aims at compelling the most recent experimental evidences indicating that changes in SR-related modulation of NMDARs also govern opposite functional dysfunctions in physiological and pathological (Alzheimer's disease) aging that finally results in memory disabilities in both cases. It also highlights SR as a relevant alternative target for new pharmacological strategies aimed at preventing functional alterations and cognitive impairments linked to the aging process.

## Introduction

Through the fine regulation of neurotransmitters/neuromodulators availability at their respective binding sites, enzymatic activities are critical for normal brain functions and are generally targeted by pathophysiological processes. In this context, the modulation of the N-methyl-D-Aspartate subtype of glutamate receptors (NMDARs) certainly represents a school case, which actually focuses the attention of a large proportion of the scientific community as illustrated by the almost 5,000 review articles referenced in pubmed. In fact, based on their large distribution throughout the nervous system and their diversity in subunit composition associated with regional specificity in the brain and even with segregated localization at synapse level (see Paoletti et al., 2013; Zhu and Paoletti, 2015), NMDARs thus appear as a perfect example to evaluate the impact of specific allosteric regulation of selective brain activities and notably of cognitive capacities, in normal and pathological conditions. These receptors are complex entities under the modulation of a wide range of regulatory processes driven by magnesium, polyamines and histamine environments as well as levels of redox state (Johnson and Ascher, 1990; Kleckner and Dingledine, 1991; Lipton et al., 1998; Choi and Lipton, 2000; Brown et al., 2001; Haas et al., 2008; Zhu and Paoletti, 2015). Beside these salient regulation features, NMDAR activation is also characterized by the obligatory fixation in addition to the main agonist glutamate of a co-agonist at a specific binding site (Traynelis et al., 2010; Paoletti, 2011; Paoletti et al., 2013). Attributed initially to glycine (Johnson and Ascher, 1987, 1992; Kleckner and Dingledine, 1988), this role of co-agonist in much brain area and particularly in those involved in cognitive functions, is now devoted to d-serine (Schell et al., 1997; Mothet et al., 2000; Snyder and Kim, 2000; Shleper et al., 2005; Billard, 2008, 2012; Henneberger et al., 2012; Bardaweel et al., 2014; Wolosker, 2018), a d-amino acid produced by the racemisation of L-serine by the enzyme serine racemase (SR) (Wolosker et al., 1999). Like the degradation of d-serine (Mothet et al., 2000; Shleper et al., 2005; Strick et al., 2011; Papouin et al., 2012; Rosenberg et al., 2013; Le Bail et al., 2015), the genetic deletion of SR impairs the connectivity and the functional plasticity of neuronal networks and has been associated with cognitive impairments (Inoue et al., 2008, 2018; Basu et al., 2009; Labrie et al., 2009; Balu and Coyle, 2012; Bai et al., 2014; Puhl et al., 2017; Balu et al., 2018). Consequently, changes in SR-dependent modulation of NMDAR activation through alterations of synaptic availability of d-serine, have been postulated to contribute to pathophysiological mechanisms governing several neurological diseases [reviewed in Billard (2013) and Coyle and Balu (2018)]. Thus, weaker NMDAR activation linked to down regulation of SR activity is now viewed as a critical synaptic dysfunction in schizophrenia, addictions, anxiety disorders, and depression (Coyle, 2006; Benneyworth and Coyle, 2012; Gómez-Galán et al., 2012; Coyle and Balu, 2018). On the opposite, up regulation of NMDAR activity due to increased production of d-serine by SR is viewed as a central mechanism for neurodegenerative processes underlying the amyotrophic lateral sclerosis (Sasabe et al., 2007; Lee et al., 2017; Kondori et al., 2018).

In the last decades, the role of SR-dependent regulation of NMDAR activity in cognitive aging has also been investigated, that is the focus of the present review. After recapitulating our knowledge that now considers NMDAR modulation by SR as an essential mechanism involved in learning and memory, currently available information related to its deregulation in physiological aging and Alzheimer's disease (AD) will be presented, with the main conclusion that a strict regulation of SR activity is required for a successful cognitive aging. This review could also offer new opportunities for considering new relevant pharmacological strategies specifically targeting the SR-associated pathway to treat memory deficits linked to age-related brain disorders.

## NMDA receptors: structure and functional regulation

**Figure 1:**
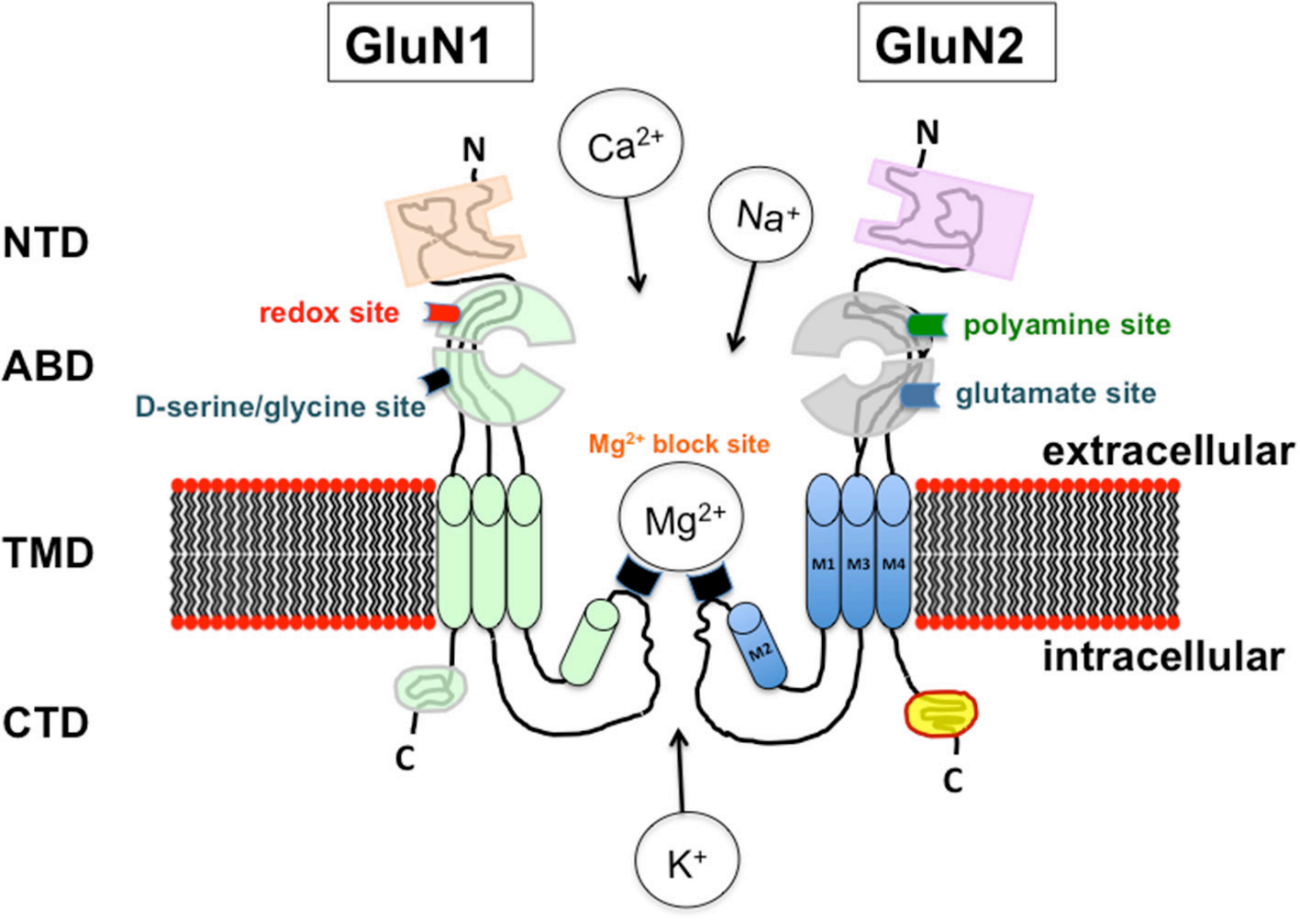
Schematic representation of the assembly and modular organization of a N-Methyl-D-Aspartic acid receptor (NMDAR). The extracellular segment includes the N-terminal domain (NTD) and the agonist binding domain (ABD) where d-serine /glycine and glutamate bind to the GluN1 and GluN2 subunit respectively. ABD also contains the redox and polyamine regulatory sites. The ion channel is localized in the transmembrane domain (TMD) that contains the site for the magnesium blockade while the C-terminal domain (CTD) is included in the intracellular segment.

NMDARs are part of a large multiprotein complex at glutamatergic synapses, that have received much attention over the last decades, due to their role in many types of neural plasticity on the one hand, and their involvement in neurotoxicity on the other hand. They are hetero-tetramers generally formed by two GluN1 subunits associated with the combination of two other partners including either four distinct GluN2 (GluN2A-D) or a mixture of GluN2 with two different GluN3 (GluN3A and 3B) subunits (Ulbrich and Isacoff, 2008; Traynelis et al., 2010; Paoletti, 2011; Paoletti et al., 2013) (Figure 1). The GluN1 subunit is expressed throughout the brain since it is mandatory for NMDAR activation through the necessary binding of a co-agonist at the amino-terminal domain of the extracellular region (Ballard et al., 2002; Paoletti et al., 2013). Besides, GluN2 subunits specifically bind the main agonist glutamate and differ from each other by their pharmacological profiles and also by providing distinct functional properties to NMDARs (Nakanishi and Masu, 1994; Dingledine et al., 1999; Hofmann et al., 2000; Paoletti et al., 2013). Although the wide range of subunit associations predicts a large diversity within the NMDARs family, preferential combinations have been regionally detected in the brain that is also observed at synaptic levels where GluN2A and GluN2B subunits are enriched at postsynaptic densities and extrasynaptic zones respectively (Traynelis et al., 2010; Paoletti, 2011; Paoletti et al., 2013). Important in the context of aging, GluN1 expression remains elevated throughout lifespan (Laurie and Seeburg, 1994; Monyer et al., 1994) whereas a progressive decrease in the GluN2B/GluN2A ratio generally occurs with age at cortical synapses (Monyer et al., 1994; Stocca and Vicini, 1998; Liu et al., 2004; Swanger and Traynelis, 2018), that have suggested the interest of pharmacologically targeting the GluN2B subunit to treat or prevent age-related memory decline (Wang et al., 2014).

In contrast to their diversity in subunit composition, all NMDARs are structurally homogenous (Figure 1) and characterized by three helices (M1, M3, M4) and a hairpin (M2) that form a transmembrane domain allowing the ion selectivity of the receptors. While this domain is subjected to tonic modulation, notably by magnesium (Mg^2+^), this is not the case for the cytoplasmic carboxy-terminal intracellular domain that controls the coupling to different intracellular signaling cascades and the receptor trafficking (Traynelis et al., 2010; Paoletti, 2011; Paoletti et al., 2013).

Compared to the other subtypes of ionotropic glutamate receptors, NMDARs display distinct functional properties identified by slow gating and deactivating kinetics associated with high calcium permeability, which depend on the subunit composition (Dunah et al., 1999; Paoletti, 2011; Wyllie et al., 2013; Zhang and Luo, 2013; Sun et al., 2017). In addition to their specific voltage-dependent blockade by Mg^2+^ (Johnson and Ascher, 1990; Kleckner and Dingledine, 1991), another impressive functional feature of NMDARs consists in their activation processes which require not only the binding of glutamate on GluN2 subunits but in synergy, the fixation of a co-agonist on a specific site present on the GluN1 components (Traynelis et al., 2010; Paoletti, 2011). This necessity of a dual binding was initially characterized in the late 80s when the induction magnitude of inward currents through native or NMDARs expressed in oocytes (Kleckner and Dingledine, 1988) or in cultured mouse neurons (Johnson and Ascher, 1987) was found to closely rely on glycine levels present in the external medium, thus revealing the existence of the so-called glycine-binding site. After more than 20 years of biochemical, immunohistochemical and electrophysiological investigations [reviewed in Billard (2012)], the initial view of glycine as the endogenous NMDAR co-agonist has then been progressively substituted by the concept assigning this role to the amino acid d-serine, though the most recent emerging view now considers that d-serine rather cooperates with glycine in a complex interplay to control NMDAR activation following time and space constraints (Mothet et al., 2015). d-serine is directly converted from its precursor enantiomer L-serine by the activity of the pyridoxal 5-phosphate (PLP)-dependent enzyme serine racemase (SR) (Wolosker et al., 1999). Interestingly, this enzyme is also able to metabolize d-serine into pyruvate and ammonia by catalyzing an α,β elimination of water (De Miranda et al., 2002; Foltyn et al., 2005). This reaction may represent an alternative route to degrade d-serine in forebrain regions where the endogenous degrading enzyme d-amino acid oxidase dAAO (Pollegioni et al., 2007), is poorly expressed (Bendikov et al., 2007; Verrall et al., 2007; Jagannath et al., 2017). However, since the efficacy of the racemisation process of L-serine is five times higher than the reaction of α, β elimination (Strísovský et al., 2005), one generally considers that SR preferentially governs d-serine synthesis.

## Serine racemase: localization, regulation and contribution to functional plasticity at synapses

**Figure 2:**
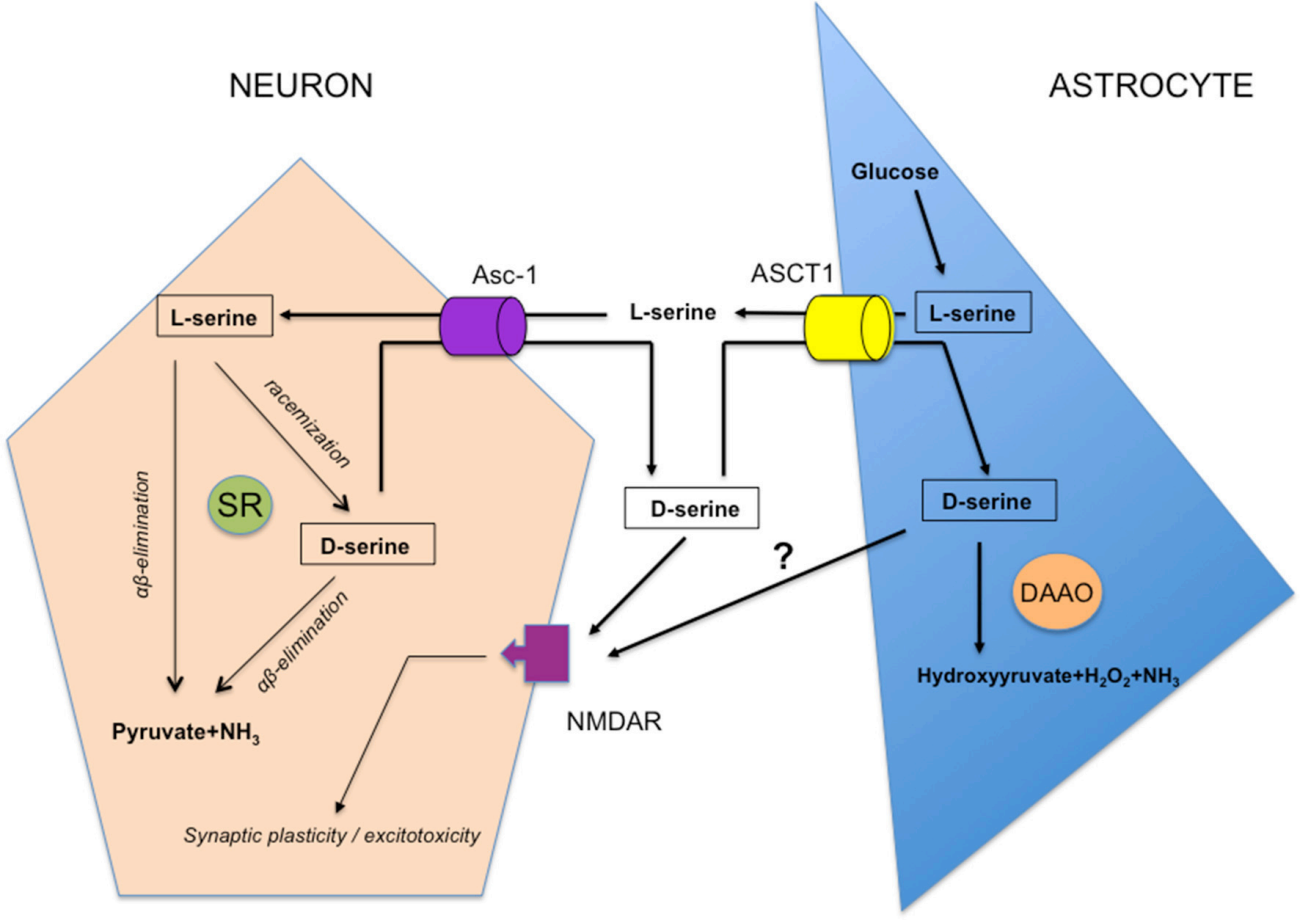
Schematic representation of the serine shuttle. L-serine specifically synthesized from glucose in the astrocyte subtype of glial cells, is released in external medium through the Alanine, serine, cysteine, threonine (ASCT1) subtype of neutral amino acid transporters. It is then taken-up by neurons through the Asc-1 subtype where it is converted into d-serine by serine racemase (SR) while part of the amino acid may be degraded into pyruvate and NH_3_ by α,β elimination of water. d-serine is delivered back to extracellular space through Asc-1 hetero-exchange with L-serine to act on NMDAR thus promoting functional plasticity at synapses or neurotoxicity in pathological conditions. d-serine is taken-up from the synaptic cleft through ASCT1 hetero-exchange with L-serine in astrocytes where it is degraded by d-amino acid oxidase (dAAO) activity. Whether part of d-serine-derived astrocytes may be released to impact NMDAR is under debate.

Nowadays, the question to know if SR is expressed in a specific cellular population at synapses is heavily discussed and has broadened to the larger debate asking if d-serine may be considered as a gliotransmitter like glutamate and ATP (Wolosker et al., 2016, 2017; Papouin et al., 2017). Indeed, the initial characterization of SR expression in astrocytes (Wolosker et al., 1999) and the view that different NMDAR-dependent functions could be driven by a vesicular release of d-serine from this subtype of glial cells (Yang et al., 2005; Panatier et al., 2006; Williams et al., 2006; Martineau et al., 2008; Papouin et al., 2012; Martineau, 2013; Lalo et al., 2018; Robin et al., 2018) are now strongly questioned. This is mainly due to the development of more selective SR antibodies and improved immunohistochemical protocols, to the lack in those pre-cited experiments of negative controls with SR knock-out (SR^−/−^) mice which display a 90% decrease in brain d-serine without significant changes in levels of the other amino acids except d-aspartate (Miya et al., 2008; Basu et al., 2009), and finally because the use of mice with disrupted SNARE-dependent exocytosis in astrocytes to specifically assess glio-transmission is still under debate (Fiacco and McCarthy, 2018; Savtchouk and Volterra, 2018). When rigorous experimental conditions are achieved *in vivo*, SR is mainly expressed in excitatory neurons and GABAergic inhibitory interneurons of the human and rodent brains with only a weak if any detection in astrocytes (Kartvelishvily et al., 2006; Miya et al., 2008; Benneyworth et al., 2012; Ehmsen et al., 2013; Balu et al., 2014; Perez et al., 2017). Nowadays, an emerging concept of a serine shuttle gathers increasing interest (Wolosker, 2011; Wolosker and Radzishevsky, 2013) in which it is viewed that through orchestrated activities of neutral amino acid transporters including at least alanine-serine-cysteine 1 (Asc-1) and ASCT1 subtypes (Rosenberg et al., 2013; Sason et al., 2017; Kaplan et al., 2018), the astrocyte-derived precursor L-serine fuels the neuronal SR to produce d-serine, which is then released to bind NMDAR before to be subsequently removed from synapses by either neurons or astrocytes (Figure 2). Although this shuttle sounds attractive to account for the synaptic turnover of d-serine in the healthy brain though it needs to be definitively validated, it fails to work when pathological conditions associated with astrogliosis prevail, such as those occurring in traumatic brain injury for example. Indeed, a controlled cortical brain insult results in a down-regulation of neuronal SR expression and a parallel increase in reactive astrocytes (Perez et al., 2017), that thus devotes a major role *in vivo* to glia-derived d-serine only when pathological mechanisms inducing excitotoxic damages and neuronal death are promoted.

In addition to help for a better determination cellular localization of SR, lessons from SR^−/−^ mice have also provided information for a pivotal role of the SR-associated processes in controlling functional plasticity at synapses. This has been particularly investigated using the electrophysiological paradigm of long-term potentiation (LTP) of synaptic transmission, a form of long lasting form of synaptic plasticity now viewed as a major functional requirement for memory formation (Izquierdo, 1991; Bear and Malenka, 1994; Collingridge and Bliss, 1995; Izquierdo and Medina, 1995; Lisman and McIntyre, 2001; Kim and Linden, 2007). Indeed, LTP is significantly reduced *ex vivo* in slice preparations isolated from mice with specific deletion of SR in neurons using the calmodulin kinase II promoter or *in vivo* using the Thy1-mediated Cre recombination, the deficits being rescued in both cases by exogenous d-serine (Benneyworth et al., 2012; Perez et al., 2017). On the contrary, similar designs but selectively targeting astrocytes using the GFAP promoter has no significant impact on LTP expression (Benneyworth et al., 2012). These results provide additional functional evidences that SR-induced d-serine from glia plays a minor role in synaptic plasticity in healthy conditions, in opposition to what is claimed (Panatier et al., 2006; Henneberger et al., 2010; Papouin et al., 2012; Lalo et al., 2018). However, it is worth noting that glia-derived d-serine could impact functional plasticity when pathological conditions prevail as recently reported after traumatic brain injury where the induction of SR expression in reactive astrocytes associated with an excessive release of d-serine, impairs LTP expression (Perez et al., 2017) and behavior (Liraz-Zaltsman et al., 2018). Whether similar deleterious effects of glia-derived d-serine on synaptic plasticity also occur in other astrogliosis-associated brain injuries remains to be determined.

The SR-dependent modulation of functional plasticity involves changes in NMDAR activation in response to altered d-serine availability. Indeed, isolated NMDAR-dependent excitatory postsynaptic currents (EPSCs) show slower decay kinetics in SR^−/−^ mice (Basu et al., 2009; Balu et al., 2013) while the amplitude of miniature NMDAR-EPSCs are significantly reduced in mice with selective neuronal SR deletion (Benneyworth et al., 2012). Providing exogenous d-serine to SR-deleted animals not only rescues these functional deficits but also increases the amplitude of NMDAR-dependent currents more extensively than in wild-type animals, consistent with lower occupancy of the NMDAR glycine-binding site when SR is invalidated.

SR is functionally modulated by a wide range of regulatory mechanisms including changes in cofactors likely to be present in the vicinity of the enzyme, protein interactions, dynamic changes in subcellular localization and posttranslational processes (recently reviewed and detailed in Wolosker, 2018). An increase in SR activity, due to activation or the prevention of its degradation, may be promoted by the small ligands ATP and Mg^2+^ (De Miranda et al., 2002; Strísovský et al., 2003; Foltyn et al., 2005), multiple protein interactors including GRIP, Golga3, Disc-1 and FBXO22 (Kim et al., 2005; Dumin et al., 2006; Ma et al., 2013; Dikopoltsev et al., 2014), by O-palmitoylation-related processes (Balan et al., 2009) and also possibly through phosphorylation at different residues (Balan et al., 2009; Foltyn et al., 2010). On the other hand, nicotinamide adeninedinucleotide (NADH) (Suzuki et al., 2015; Bruno et al., 2016), protein interactions with Pick-1 (Fujii et al., 2006), PSD-95 (Ma et al., 2014; Lin et al., 2016), SAP102 and stargazin (Ma et al., 2014), membrane or nuclear translocations (Balan et al., 2009; Kolodney et al., 2015) and S-Nitrosylation-related oxidative processes (Mustafa et al., 2007) inhibit SR activity. Therefore, the SR activity itself appears to be modulated in a complex manner by a large mosaic of mechanisms, which can be targeted by the aging process.

## Down regulation of SR-related activity in physiological AGING

**Figure 3:**
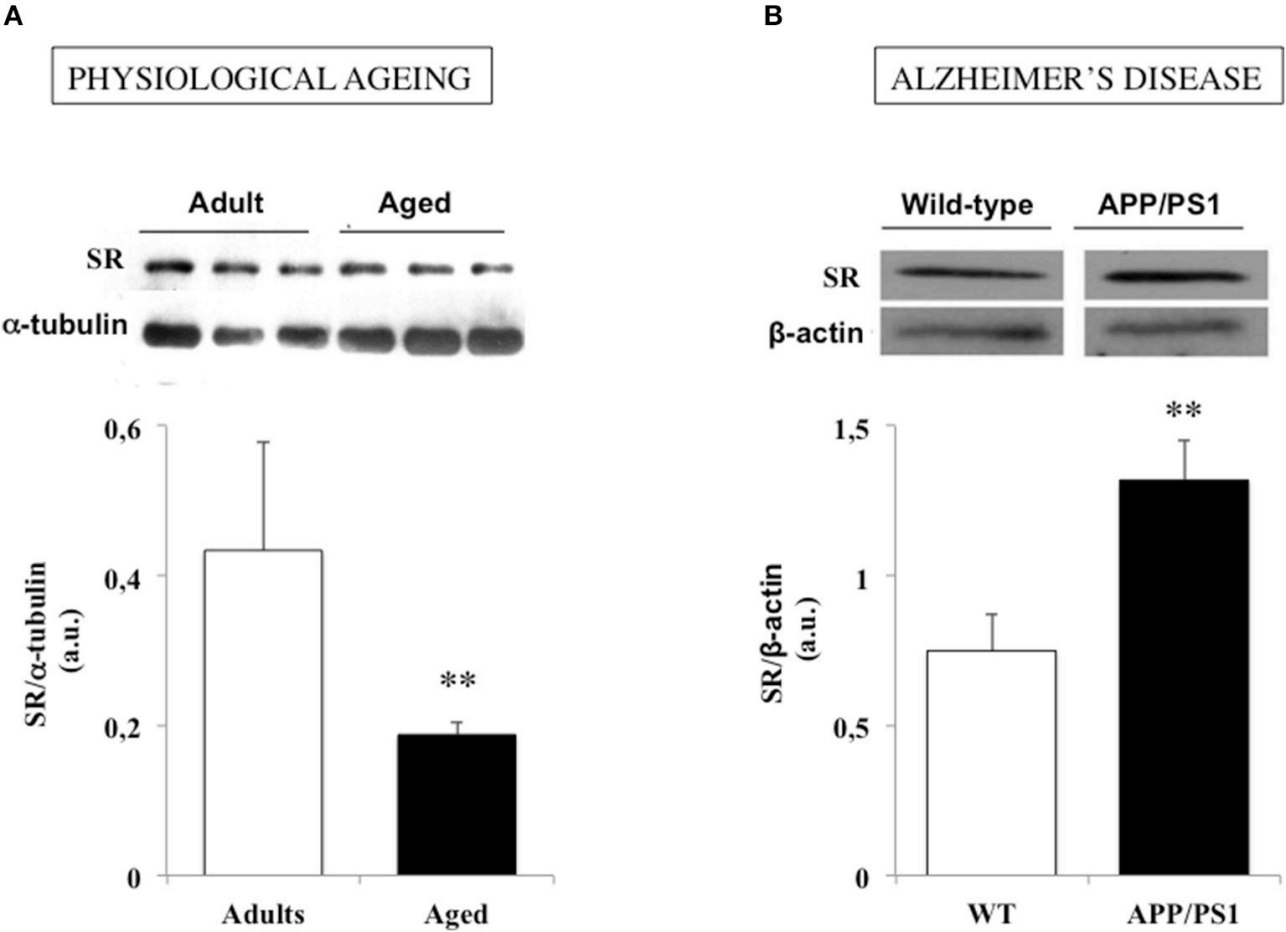
Serine racemase (SR) expression is down and up regulated in physiological and pathological brain aging respectively. **(A)** Examples of immunoblots for serine racemase (SR) and α-tubulin in adult and aged rats (up) and bar graphs depicted the mean SR expression determined for each group when normalized to α-tubulin (down). **(B)**. Examples of immunoblots for SR and β-actin in a wild-type (WT) and an APP/PS1 mouse model of Alzheimer's disease (up) and bar graphs depicted the mean SR expression determined for each group when normalized to β-actin (down). (**P < 0.01). Modified with permissions from (Potier et al., 2010) and (Madeira et al., 2015).

Changes in neurologic functions generally occur with physiological aging that may substantially interfere with everyday activities (Craik and Bialystok, 2006). Indeed, older adults experience deficits in learning and memory while the speed of cognitive processing is frequently slowed down, that have initially been associated with neuroanatomical changes (Brunso-Bechtold et al., 2000; Driscoll et al., 2003; Finch, 2003; Geinisman et al., 2004; Hayakawa et al., 2007; Burke and Barnes, 2010). However, lessons from numerous preclinical investigations now rather support the view that impaired expression of NMDAR-dependent functional plasticity at synaptic connections is the major cellular substrate of physiological cognitive aging (Lynch, 1998; Barnes, 2003; Billard, 2006; Foster, 2012). A decrease in NMDAR density, and notably in GluN2B subunits, was initially suspected to underlie LTP deficits in the aging brain (Magnusson, 1998, 2000; Clayton et al., 2002a,b; Magnusson et al., 2002; Bai et al., 2004; Brim et al., 2013) but defects affecting the functional modulation of the receptor have also been later characterized including deregulation at the redox site (Kuehl-Kovarik et al., 2003; Bodhinathan et al., 2010; Yang et al., 2010; Kumar et al., 2017), changes in non-competitive blockade (Norris and Foster, 1999) and even altered lipid composition of postsynaptic membranes (Lynch and Voss, 1994; McGahon et al., 1999; Latour et al., 2013). In the search of such functional deficits, changes in SR-modulation of NMDAR activation has also been postulated to develop with age (Billard, 2013). According to this possibility, aged humans with impaired memory capacities in the Groton maze computer test improve their performances if they previously receive a d-serine-enriched drink (Avellar et al., 2016) while learning deficits in aged drosophila in an olfactory conditioning is rescued by feeding the flies with the amino acid (Yamazaki et al., 2014). Subsequent analyses in aged rodents indicate that a reduced SR expression is a prominent feature of hippocampal aging (Figure 3A), which decreases d-serine levels within neuronal networks and promotes NMDAR hypofunction (Mothet et al., 2006; Potier et al., 2010; Turpin et al., 2011). Providing the amino acid to the “aged” tissues then restores NMDAR activation and LTP induction at synapses (Yang et al., 2005; Mothet et al., 2006; Turpin et al., 2011). In animal models of successful cognitive aging such as the LOU/C strain of rats (Alliot et al., 2002; Kappeler et al., 2004), the potent memory abilities and NMDAR-dependent LTP displayed by aged individuals correlate with preserved SR expression and d-serine production (Kollen et al., 2010; Turpin et al., 2011). One characteristic of aged LOU/C rats is to present high resistance to oxidative stress (OS) induced by the accumulation of free radical damages that progressively take place in the course of aging (Sohal and Weindruch, 1996; Golden et al., 2002; Ali et al., 2006; Dröge and Schipper, 2007). Increased oxidation of sulfydryl groups of SR (Mustafa et al., 2007) and/or changes in its dimer active conformation (Wang and Barger, 2012) could then be viewed as critical mechanisms driven by OS to impact SR activation in the aging brain. Accordingly, long-term treatment with the reducing agent N-acetyl cysteine in aged rats to prevent from OS development, protects SR expression and activity and preserves a potent NMDAR activation in the animals (Haxaire et al., 2012). In addition, weaker SR activity promoted by OS could also be managed through an hypermethylation in the promoter of SR gene (Zhang et al., 2015) that could explain the age-related decreased levels of SR transcripts (Mothet et al., 2006; Potier et al., 2010). These results therefore reinforce the idea of preventing oxidative stress as a major strategy to alleviate cognitive aging (Sohal and Weindruch, 1996; Liu et al., 2003; Dröge and Schipper, 2007).

Besides the OS-dependent dysfunctions of SR activation, a down-regulation of its enzymatic activity could also be viewed in the aging brain as resulting from a reduced synaptic availability of L-serine (postulated in Ivanov and Mothet, 2018). However, though the expression of PHGDH, one of the enzymes predominantly involved in the synthesis pathway of the d-serine precursor (Yamasaki et al., 2001), is reduced in acutely isolated astrocytes from aged mice (Orre et al., 2014; Holtman et al., 2015), overall levels of the amino acid are not altered in the aging hippocampus (Mothet et al., 2006; Turpin et al., 2011; Haxaire et al., 2012) and providing L-serine does not help in preventing the age-related decrease in NMDAR activation (Junjaud et al., 2006). On the other hand, recent evidence reports that the D-serine shuttle, and notably the potency of the Asc-1 transporters to release D-serine from neurons, is not affected by age (Billard and Freret, 2018). These results further indicate that changes in SR-related modulation of NMDAR represent a critical mechanism associated with physiological brain aging and that boosting SR activation could thus be viewed to represent an alternative strategy to alleviate age-related memory impairment. Among different possibilities, a strategy based on SR stimulation by Mg^2+^ could be hypothesized considering that Mg^2+^ has been shown to enhance learning and memory (Ozturk and Cillier, 2006; Slutsky et al., 2010).

## Up regulation of SR-related activity in Alzheimer's disease

Compared to other neurological disorders such as schizophrenia, depression or amyotrophic lateral sclerosis (Goltsov et al., 2006; Labrie and Roder, 2010; Mitchell et al., 2010; Gómez-Galán et al., 2012; Balu and Coyle, 2015; Coyle and Balu, 2018), our current knowledge on the role of the SR-related pathway in the pathophysiology of Alzheimer's disease (AD) is so far limited. One reason for this weaker interest probably comes from the initial biochemical observations indicating that free d-serine levels were not consistently altered in the brain of AD patients, although the percentage of d-serine in the total (d + l) serine was significantly lower than that of aged-matched controls (Chouinard et al., 1993; Kumashiro et al., 1995; Nagata et al., 1995; Hashimoto et al., 2004; Biemans et al., 2016) but see (Fisher et al., 1998). Nevertheless, the absence of a clear-cut contribution of SR to AD-related pathophysiology could reflect the fact that the levels of d-serine in those experiments were determined in patients at late stages of the pathology whereas the most recent preclinical studies suggest that the amino acid could rather be involved in the very early steps of the disease (Madeira et al., 2015). Indeed, a significant increase in d-serine levels has recently been characterized in the cerebrospinal fluid (CSF) of subjects with only mild cognitive impairment that will probably evolve into dementia (Madeira et al., 2015). This observation has suggested that a deregulation of the SR-related activity could serve as a new biomarker of the entry into the pathology [see also (Hashimoto et al., 2004)], although this postulate has not recently been confirmed (Biemans et al., 2016). Nevertheless, several preclinical data strongly argue for the involvement of SR in the pathophysiological processes underlying AD. Thus, two major soluble factors involved in AD pathogenesis, the amyloid ß-peptide (Aß) and the secreted form of ß-amyloid precursor protein (APP) (Cline et al., 2018), stimulate SR expression and promote d-serine release in microglial cell cultures whereas these subtypes of glial cells do not normally produce the amino acid (Wu et al., 2004, 2007). The Aß peptide evokes d-serine synthesis and efflux also from neurons, in synergy with the release of glutamate (Brito-Moreira et al., 2011; Madeira et al., 2015) that drives over-stimulation of NMDAR and promotes neurotoxicity, a typical picture of the pathophysiology of AD (Harkany et al., 2000; Butterfield, 2002; Hynd et al., 2004). Several other preclinical observations fit well with a contribution of d-serine in AD-related neurotoxicity: neuronal cell death induced by NMDA is strongly reduced in cerebral tissues depleted in d-serine after a pre-treatment with dAAO (Katsuki et al., 2004) as well as in organotypic hippocampal slices pre-treated with the recombinant d-serine deaminase, an enzyme 100 fold more active than dAAO in degrading the amino acid (Shleper et al., 2005). *In vivo*, both NMDAR and Aß-induced neurotoxicity are largely attenuated in SR^−/−^ mice (Inoue et al., 2008). Through the binding of inducible proto-oncogenes *c-*fos and JunB to the activator protein-1 sequence present on the first intron of the SR gene, Aß promotes the transcriptional induction of SR (Wu and Barger, 2004), an observation which fits with the increase in SR messenger RNAs in the brain of AD patients (Wu et al., 2004). Post-transcriptional mechanisms may also contribute such as an increase in intracellular calcium levels by Aß (Wu et al., 2004) knowing that calcium overload in neurons is able to boost SR activity (Cook et al., 2002; De Miranda et al., 2002).

Besides, a significant increase in SR expression and d-serine levels also occur *in vivo* in a mouse model of AD with a transgene for APP associated with a mutant form of presenilin 1 (APP/PS1 mice) (Madeira et al., 2015). Finally, recent preliminary data indicate that in the 5xFAD model of AD which expresses high levels of soluble Aß oligomers (Oakley et al., 2006; Giannoni et al., 2013; Lee and Han, 2013), the impaired functional plasticity reported at hippocampal synapses (Kimura and Ohno, 2009; Crouzin et al., 2013) was rescued after deleting the SR gene, that further points out a major role of an altered SR-dependent modulation of NMDAR functions in the Aß-related pathophysiology of AD (Billard et al., 2018).

Considering the current state of knowledge summarized above, the elevated SR expression and the subsequent increase in d-serine levels in the extracellular space could be viewed as pro-death signals in AD that promotes, in conjunction with the release of glutamate, the neurotoxicity exhibited by inflammatory processes (Barger et al., 2007; Vesce et al., 2007). Although this view clearly remains to be definitively characterized and notably if the glia-derived SR could contribute to mechanisms of the insult, the up-regulation of the SR-related pathway in AD therefore appears as a perfect example of how a deregulation of allosteric modulation of NMDAR may drive the onset of pathological conditions.

## Conclusion

**Figure 4:**
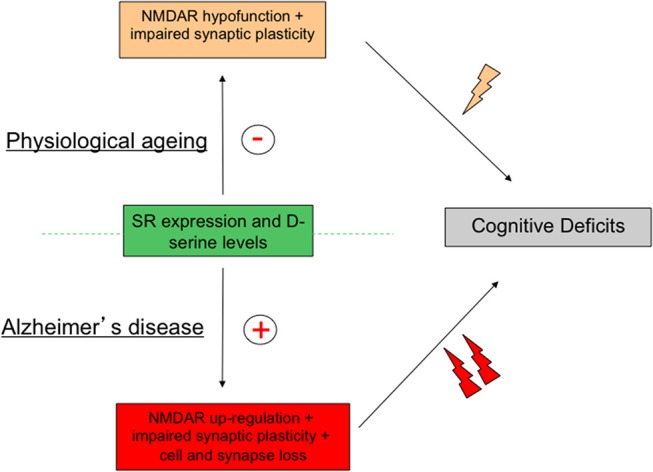
Activity of serine racemase (SR) must be strictly regulated to avoid age-related memory deficits. Schematic diagram outlying the concept that although changes in SR expression and activity are opposite in physiological and pathological brain aging brain through down- and up-regulation of N-Methyl-D-Aspartic acid receptor (NMDAR) activity respectively, cognitive deficits, and notably memory impairments, represent the ultimate syndrome in both conditions.

Nowadays, a wealth of preclinical and clinical evidences argues for a critical role of SR throughout lifespan in the regulation of functional plasticity through the synaptic availability of the NMDAR co-agonist d-serine. Such modulation impacting NMDAR activation allows the enzyme to control many brain functions in healthy conditions while being a preferential target for pathophysiological insults (Coyle and Balu, 2018). When interest is focused on age-related memory disabilities, a down- and up-regulation of the SR-associated pathway are specifically associated with physiological aging and AD respectively. Although these alterations show striking opposite directions, they both result *in fine* in memory deficits indicating that a strict control of SR expression and activity is required to achieve a successful cognitive aging (Figure 4). These results therefore highlight SR as a potent target for the development of alternative pharmacological interventions aimed at relieving cognitive impairments linked to the aging process. Protection of SR to the age-related oxidative stress is already suggested to represent such an alternative procedure to rescue memory deficits associated with physiological aging (Haxaire et al., 2012). In preclinical studies, SR antagonists such as Phenazine Ethosulfate (Phen-Et) and erythro-β-Hydroxy L-aspartate have been used to investigate SR involvement in specific NMDAR-dependent processes (De Miranda et al., 2002; Kim et al., 2005; Strísovský et al., 2005; Stevens et al., 2010), that could represent other pharmacological alternatives to prevent the onset of pathological conditions in which SR activity is facilitated such as ALS, AD or brain trauma (Sasabe et al., 2007; Madeira et al., 2015; Lee et al., 2017; Perez et al., 2017; Kondori et al., 2018), though the specificity of these pharmacological tools have recently been questioned. However, there is no doubt now that increasing our knowledge of SR-dependent regulation of NMDAR activation certainly represents a key route that will help people keeping potent cognitive abilities throughout lifespan.

## Author contributions

The author confirms being the sole contributor of this work and has approved it for publication.

### Conflict of interest statement

The author declares that the research was conducted in the absence of any commercial or financial relationships that could be construed as a potential conflict of interest.
